# Three-dimensional nanostructure analysis of non-stained Nafion in fuel-cell electrode by combined ADF-STEM tomography

**DOI:** 10.1093/jmicro/dfae002

**Published:** 2024-01-13

**Authors:** Takuji Ube

**Affiliations:** Nano-scale Characterization Center, JFE Techno-Research Corporation, Kawasaki-Ku, Kawasaki City, Kanagawa, Tokyo 2100855, Japan

**Keywords:** PEFC, tomography, 3D, STEM, segmentation, ionomer

## Abstract

The polymer electrolyte fuel cell (PEFC) is one of the strongest candidates for a next-generation power source for vehicles which do not emit CO_2_ gas as exhaust gas. The key factor in PEFCs is the nano-scaled electrochemical reactions that take place on the catalyst material and an ionomer supported by a carbon support. However, because the nano-scaled morphological features of the key materials in the catalyst compound cannot be observed clearly by transmission electron microscopy, improvement of PEFC performance had been approached by an imaginal schematic diagram based on an electrochemical analysis. In this study, we revealed the nano-scaled morphological features of the PEFC electrode in three dimensions and performed a quantitative analysis of the nanostructure by the newly developed ‘Combined ADF-STEM tomography technique’. This method combines information from plural annular darkfield detectors with different electron collection angles and can emphasize the difference of the electron scattering intensity between the ionomer and carbon in the cross-sectional image of the reconstructed three-dimensional (3D) data. Therefore, this segmentation method utilizing image contrast does not require a high electron beam current like that used in energy dispersive X-ray analysis, and thus is suitable for electron beam damage-sensitive materials. By eliminating the process of manually determining the thresholds for obtaining classified component data from grayscale data, the obtained 3D structures have sufficient accuracy to allow quantitative analysis and specify the nano-scaled structural parameters directly related to power generation characteristics.

## Introduction

The phase-out of fossil fuel combustion vehicles has become a global trend in the 2020s, but in order to prevent further global warming, next-generation power sources that do not emit CO_2_ exhaust gas will be required [1,2]. The polymer electrolyte fuel cell (PEFC) [3] is a promising power source with the potential to become an alternative to the internal combustion engine (ICE), on the same level as battery electric vehicles (BEVs). The key part of electric generation by a fuel-cell system is the electrochemical reactions that take place on the surface of a catalyst metal surrounded by an ionomer which is supported by carbon black. Because the nanostructure of these PEFC components, and especially the three-phase interface of the components, plays the role of transporting gases (hydrogen, air and water vapor), protons and electrons, the nanostructure of these components significantly affects PEFC performance [4,5]. Furthermore, since low cost is also important for practical application, the developers of PEFC power sources strongly require optimization of the use of Pt [6] and reduction of the amount used [7].

The scanning transmission electron microscope (STEM) is one of the most powerful tools in material science for observation with high spatial resolution in various types of analyses using a finely converged electron beam. However, two extremely difficult problems arise in quantitative observation of these PEFC nanostructures by STEM: One is the poor electron resistivity of ionomers [8], and the second is the very small difference in contrast between the ionomer and the carbon support due to their similar densities and atomic numbers. To overcome this problem, previous reports on (S)TEM analysis of ionomers including PEFC catalysts at the single-nanometer level have examined the staining method [8–11], cryogenic technique [12,13] and freehand segmentation [14–16], but neither observation of a unmodified PEFC catalyst nor an elimination of arbitrariness has been established. Here, we propose an analysis technique for a nanoscaled unmodified PEFC catalyst by combined annular darkfield scanning electron microscope (ADF-STEM) tomography. We also developed an arbitrariness-free segmentation recipe for extracting binary images of materials and demonstrated quantitative evaluation using reconstructed 3D data, especially for the fraction of ionomer versus the carbon support.

## Methods

### Sample preparation

In this study, a commercially available catalyst powder for PEFCs, TEC10E50E (Tanaka Kikinzoku Kogyo K.K., Japan) was used. The Pt nanoparticles were supported on a high-specific surface area carbon with a 50 wt% ratio of the catalyst (Pt) to the support (C). The ionomer used here was Nafion D2020 (DuPont, USA).

The prepared slurry mixture of the catalyst and ionomer was spray-coated on a transfer sheet of polytetrafluoroethylene (PTFE), and the catalyst carrying rate was controlled to 0.3 mg· cm^−2^. The coated slurry films were transferred to a Nafion electrolyte membrane (NR211 (DuPont, USA)) by hot pressing. Because the samples for TEM observation were prepared by the scraping method from the hot-pressed catalyst layer, the samples maintained the same aggregation state as the power generation state of the PEFC. Here, two samples with different mixing composition ratios (I/C) of 0.5 and 2.0 were prepared.

### Electron microscopy data acquisition

The electron microcopy experiments were performed using a Talos F200X (Thermo Fisher Scientific Inc., USA) equipped with a quad windowless energy dispersive X-ray (EDX) detector system (Super-X). The acceleration voltage of the incident electron beam was 200 kV. EDX spectral imaging was performed for 20 μs as the dwell time. More than 300 frames of images were acquired to ensure a high signal-to-noise ratio, and also to suppress damage of the ionomer. Three ADF detectors were inserted on the optical axis of the penetrating electron beam, and their scattered electron collection angles were set as shown in Table 1 by controlling the camera length of a STEM instrument parameter.

**Table 1.** Electron collection angles of STEM detectors (unit: mrad)

Detector	Inner angle	Outer angle
HAADF	37	200
MAADF	14	35
LAADF	8	12
BF		6

Combined ADF-STEM tomography was performed using Tomography STEM software (Thermo Fisher Scientific Inc., USA). Series tilt images were taken from −78° to +80° at 1° intervals. The STEM images were acquired using the three ADF detectors and one BF detector with 2 048 × 2 048 px resolution and a pixel size of 0.183 nm. The reconstruction area was cropped to 1 024 × 1 024 px from the aligned image stack, and the finally reconstructed field of view was 187 nm cubed.

### 3D-reconstruction and segmentation

The obtained series tilt image stacks were aligned by two steps of the cross-correlation method and bead tracking method in order to reconstruct the most accurate tomogram possible. These image alignment calculations were performed only for the HAADF image stacks, because HAADF shows clear contrast of the Pt nanoparticles due to its large Z (atomic number). The calculated image shift values were then applied to the other image stacks.

3D-reconstruction calculation was performed for all aligned images stacks by the expectation-maximization (EM) method for 20 iterations. To exclude low-spatial resolution areas in the 3D images, the reconstruction area was cropped to 1 024 × 1 024 px around the center of the image stack. The alignment and 3D-reconstruction processes were performed on Inspect 3D software (Thermo Fisher Scientific Inc., USA).

**Fig. 1.**
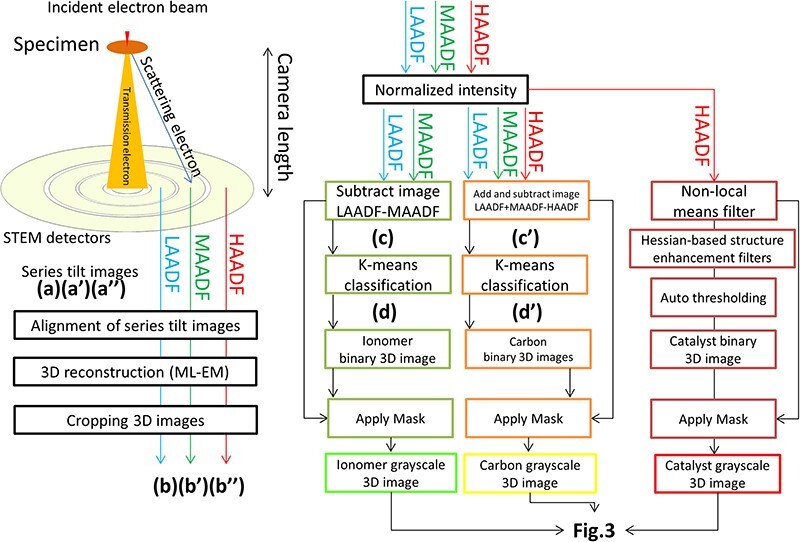
Scheme of combined ADF-STEM tomography. The intermediate images during processes (a) to (d’) are shown in Supplemental data 1 (Supplementary Fig. S1).

The scheme in Fig. 1 shows the indicated image segmentation process for reconstructing 3D images of the respective materials, i.e. the carbon support (C), ionomer (F) and catalyst particles (Pt). Avizo software (Thermo Fisher Scientific Inc., USA) was used in this image segmentation and quantitative calculation.

## Results

### 2D STEM images and EDX element mapping images

**Fig. 2.**
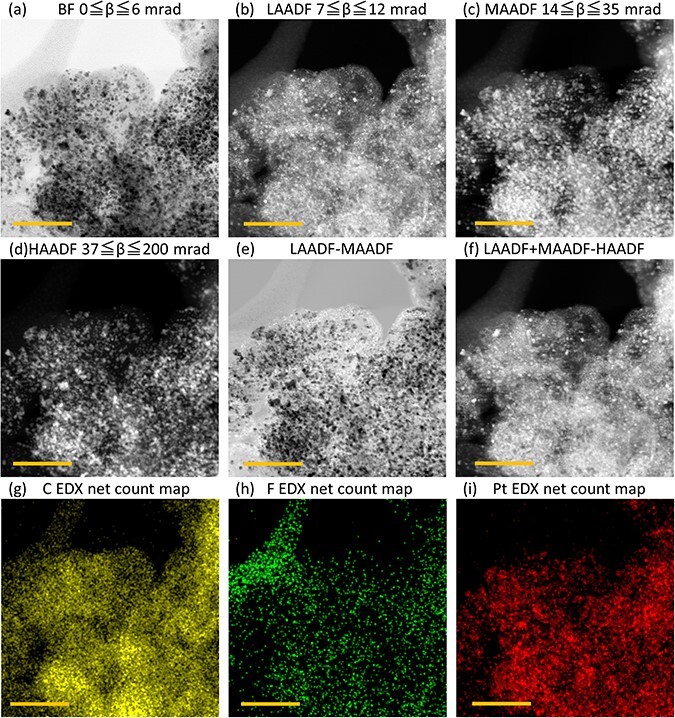
2D-STEM images of catalyst in PEFC electrode. (a)–(d): Raw STEM images, (e)–(f): calculated STEM images, and (g)–(i) EDX elemental mapping images. All scale bars indicate 50 nm. The brightness and contrast of all images were corrected by auto function by Velox software (Thermo Fisher Scientific Inc.).

Figure 2 shows the representative STEM images of the catalyst in the PEFC electrode, including EDX spectroscopic elemental mapping images. First, the EDX elemental mapping images of C and F ((g) and (h)) could distinguish individually between the carbon support (C) and the ionomer (F), and their 2D spatial distributions were expressed. The quad EDX detectors and high-speed beam scanning made it possible to analyze the spatial distribution of the ionomer before it disappeared due to electron beam irradiation without using the cryogenic technique [17].

The three ADF-STEM images ((b)–(d)) show a similar bright- contrast proportional to mass and thickness. However, the difference in the collection angle range of the scattering electrons indicates significant distinctions in contrast. The LAADF image (b) shows clear contrast of the ionomer and the carbon support. In particular, these results confirmed the correspondence at the bridge-like contrast in the upper-left corner, which is also shown as high EDX counts in the elemental mapping image of F (h). The round shape of the carbon-support particle is also observed clearly, and its high-crystallinity regions show glittering contrast due to the Bragg–diffracted electrons of (00 · 2), (01 · 0) and (01 · 1) from the graphite crystal incident to the LAADF detector. Likewise, the Pt (111) Bragg–diffracted electrons also entered the LAADF detector, and some Pt particles showed bright contrast. The two ADF images acquired by the MAADF and HAADF (Fig. 2c and d) indicate a generally similar Z-contrast, and there was only a slight difference in contrast between the carbon and ionomer. These four STEM images (Fig. 1a**–**d) were obtained simultaneously, and each pixel gives information from the same specimen position. Arithmetic operation of these images could either enhance or diminish the contrast; for example, the subtraction image of LAADF–MAADF (Fig. 1e) would show a clear contrast between the component elements. However, it was not possible to distinguish the ionomer and carbon support inside the primary particle of the catalyst in contrast due to the thickness of the specimen.

### 3D-reconstructions of element mapping images by combined ADF-STEM tomography

STEM tomography is a useful tool for revealing the 3D spatial distribution of nanostructured materials. The tomography technique also provides not only a 3D image visualization but also cross-sectional 2D images in the same way as X-ray computer tomography images. All cross-sectional slices have identical thicknesses, and the single cross-sectional ADF-STEM images substantially correspond to the scattering potential distribution on the cross-sectional image plane. Therefore, cross-sectional imaging analysis is able to distinguish the constituent elements by signal intensity, i.e. Z-contrast without the influence of thickness. As mentioned earlier, arithmetic operations between plural ADF images can be expected to enhance the contrast of light elements such as C and F, corresponding to the carbon support and ionomer, respectively.

**Fig. 3.**
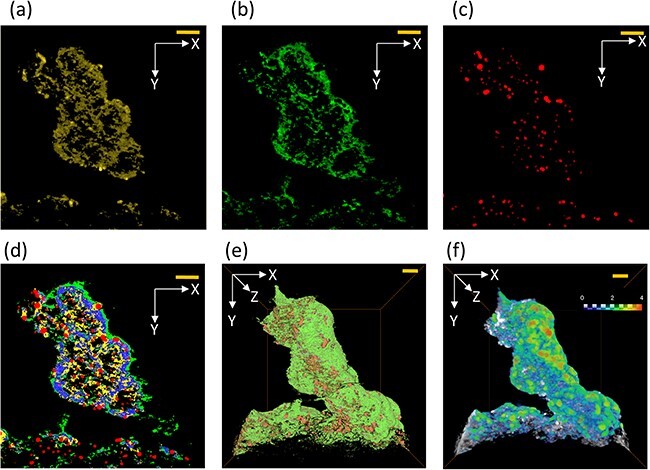
Cross-sectional 2D images and 3D-rendered images (I/C = 2.0). (a): Carbon support, (b): ionomer, (c): Pt, (d): composed images (yellow: carbon support without impregnated ionomer, blue: carbon support with impregnated ionomer, green: ionomer without impregnation to the carbon support, red: catalyst), (e): 3D-rendered image, and (f): 3D-thickness map of ionomer. (The color bars in the figure correspond to the film thickness of the ionomer from 0.2 nm (blue) to 4 nm (red)). All scale bars correspond to 20 nm.

Figure 3 shows the extracted cross-sectional segmented 2D images ((a), (b), (c), (d)) from the 3D structure and a 3D rendering image (d). All cross-sectional 2D images were generated by combined ADF-STEM tomography without EDX analysis and were the calculated data from the different combinations of ADF detectors (Fig. 1). Therefore, each elemental image has an individual spatial distribution, and the overlapping information between images can be expressed as shown in (d). The blue region in Fig. 3d expresses the overlapping of the carbon support and ionomer. Since the spatial resolution of tomography is limited to ∼1–2 nm by the Crowther criterion [18] and/or image alignment accuracy, small pores in carbon support cannot be expressed by tomography. However, the ionomer can impregnate into these small pores, and it has been expressed as an overlap on the carbon support. This impregnation of the ionomer into the carbon support will be discussed in the following section. The 3D rendering image (e) reveals the coverage of the ionomer on the carbon support. Although this coverage state was important in conventional PEFC catalysts which used a solid carbon support such as Vulcan, modern PEFC catalysts use a carbon support with high-specific surface area, which means the apparent coverage ratio is less important. Nevertheless, the distribution of the ionomer coating thickness is directly related on transport phenomena and is considered to be a parameter that affects the power generation characteristics of PEFCs. This method enables visualization and statistical evaluation of the ionomer thickness distribution (e.g. see Fig. 3f). The detailed results are discussed in the following section.

Table 2 shows the 3D quantification results in this study, comparing the catalysts with the two different I/C ratios of 0.5 and 2.0. The qualitative tendencies were summarized in Table 2, in which the I/C ratio (calculated) corresponds to the I/C preparation ratio. However, the impregnation ratio decreased at the higher I/C ratio, indicating that a relatively larger amount of ionomer had adhered outside the carbon support. Since this report focuses on the separation and visualization of the ionomer and carbon in 3D, the details will be reported in a following report. The sphere equivalent diameter and coordinates of each Pt particle can also be analyzed. Here, it should be noted that quantitative evaluation of the position of catalyst particles on the carbon support [19] and the relative distance between the ionomer and carbon/catalyst is also possible.

**Table 2.** 3D quantification results

	I/C: 0.5		I/C: 2.0	
	Volume	Area	Volume	Area
	nm^3^	nm^2^	nm^3^	nm^2^
Carbon	8.5E + 05	1.2E + 06	2.76E + 05	5.89E + 05
Total ionomer	5.5E + 05	1.3E + 06	4.77E + 05	1.03E + 06
Inside ionomer	5.0E + 05	1.2E + 06	1.96E + 05	5.05E + 05
Outside ionomer	5.2E + 04	4.8E + 05	2.80E + 05	1.05E + 06
I/C (wt) (calculated)	0.75		2.02	
Impregnation ratio (%)	90.6		41.2	

## Discussion

### Difference in contrast information due to electron collection angle in ADF detectors

In general, in ADF-STEM images (e.g. HAADF-STEM images), the signal intensity depends on approximately the square of the atomic number (Z) of a single atom or amorphous material. Although Yamashita *et al*. reported that this power law will be closer to 2.0 for higher-scattering angles [20], the power law cannot be substantiated for light elements and low-scattering angles because the atomic scattering factor dominates in the low-scattering angle region.

**Fig. 4.**
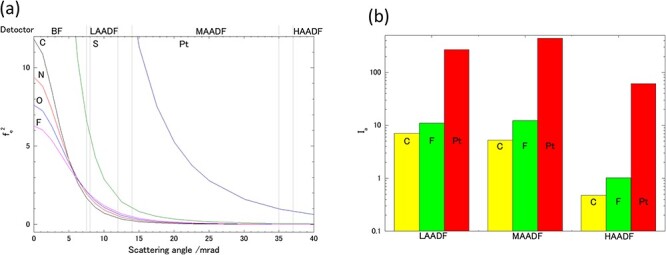
(a): Squares of atomic scattering factors for an electron beam (200 kV), (b): calculated incident signal intensities of each ADF detector used in this study.

Figure 4a shows the relationship between the squares of the atomic scattering factors for an electron beam (200 kV) of the region of interest elements in this study [21] and the collection angles of the ADF detectors used here.

In the LAADF-STEM images, the major signal intensity is dominated by elastic scattering.

The incident ADF signal intensity *I_a_* by scattering from an amorphous substance is approximately described by the following Eq. (1).


(1) $${{\mathrm{I}}_a} = \smallint \limits_{{\beta _i}}^{{\beta _o}} 2\pi \beta f{\left( \theta \right)^2}d\beta $$

Where ${\beta _i}$ and ${\beta _o}$ are the respective inner collection angles and outer collection angles of each ADF detector, and $f{\left( \theta \right)^2}$ is the square of the function of the atomic scattering factor. Figure 4b shows the results of a calculation in which the actual values of the equipment conditions used in this study were substituted into Eq. (1). The contrast of the actual ADF-STEM images (e.g. Fig. 2b**–**d) corresponds to the scattering potential of the specimen in the range of collection angles for scattering electrons. That is, a bright contrast indicates the existence of scattering, and its intensity increases linearly with the number of atoms and/or atomic number at the beam scanning position.

According to results in Fig. 4b, an ADF detector was set to a high-angle arrangement (HAADF) and the gain and offset of the amplifier were set were set to levels that would not cause saturation of the Pt signal (contrast), resulting in an incident signal of carbon (C) and ionomer (F) of about 1/150 to 1/60. Modern commercially available STEM systems have sufficient sensitivity to detect single-electron counts [22–24], but their linearity and dynamic range of output intensity for incident electron intensity are still inadequate to distinguish a three-order digit difference in incident intensity [25–27]. Therefore, in theory, it is not possible to distinguish the ionomer and carbon by HAADF-STEM observation without saturation of the Pt signal, and signal saturation then results in an incorrect tomogram in STEM tomography. On the other hand, under the LAADF conditions, carbon (C) and the ionomer (F) have intensity ratios of about 1/40 to 1/25, respectively, so the acquired signal is within the dynamic range of the detection system relative to the incident electron beam intensity without saturation. In the MAADF detector, the Pt/C and Pt/F intensity ratios are within two orders of magnitude, which is an intermediate level between the HAADF and LAADF conditions. The C/F intensity ratio is also the largest among the three ADF detectors. In this case, the actual intensity ratio of the carbon support and ionomer is expected to be even lower than the result of this simple calculation because the ionomer used in PEFC catalysts is composed by a CF_2_ bond in its backbone structure. Therefore, in this study, ideal pure Z-contrast images within a slice (0.18 nm thick), which is too thin to exist in reality, were extracted by tomographic calculation, and the contrast between the carbon and the ionomer was further enhanced by image calculation with different ADF detector signals to improve the accuracy of automatic segmentation using the K-means method. The brightness of the Pt particles in the 2D images acquired by the LAADF and MAADF detectors is affected by Bragg reflection, which means that the brightness is not in correct Z-contrast. In principle, the sample-series tilt averages the excitation conditions of Bragg reflection, but it does not give a correct tomogram of Pt particles. We solved these problems by using calculated LAADF and MAADF images to identify the carbon and ionomer, and only HAADF images, which have the largest Z-contrast contribution, to identify Pt particles, overriding the incorrect Pt information given by the LAADF and MAADF images described earlier.

Another major advantage of the method developed in this study is the large intensity of the scattering electrons in the LAADF and MAADF regions. As a tradeoff for the problems of Bragg reflection, etc., the incident electron intensity of light elements is about 10 times higher than that of the HAADF region, so sufficient contrast can be obtained even if the amount of electron irradiation of the sample is drastically reduced.

### Acquisition of series tilt STEM images and 3D reconstruction calculations

Several advantages of STEM(-ADF) tomography compared to TEM(-BF) tomography have been reported [28,29]. In particular, STEM(-ADF) tomography is advantageous for analysis of thick specimens as it is not necessary to consider contrast degradation due to an inelastic scattering electron beam and the contrast transfer function of objective lens, i.e. phase contrast. We obtained 159 series tilt images by the STEM method up to a relatively high angle of ∼±80° in order to minimize the missing wedge of a reconstruction calculation. The coefficient *T* for the penetration thickness of the electron beam depending on the tilt condition is the proportion given by $T = 1/cos\theta $ for the tilt angle θ. The relative thickness of a conventional slab-type specimen under an 80° tilt condition is 5.8 times thicker than that under the 0° condition. When high-tilt angle images for slab-like specimens are included, for example, in the case of a cupping artifact, even STEM tomography cannot obtain meaningful images for 3D reconstruction because of the non-linear signal intensity relationship with increasing thickness [30]. Therefore, we selected the standard Cu TEM grid with a microgrid carbon film, and a field of view in which the specimen protruded into the vacuum from the carbon grid and was not affected by the shadows of the grid or sample holder, even at the maximum tilt angle. Since the observed carbon support aggregates were fitted at the center of the 3D reconstruction area (187 nm^3^) with room to spare, the maximum penetration thickness was ≤100 nm, which is independent of the specimen tilt angle. Furthermore, the depth of focus field ${\Delta}z$ is important for obtaining an accurate 3D reconstruction calculation. ${\Delta}z$ can be written as [31]


(2) $${\Delta}z = \frac{{1.7\lambda }}{{{\alpha ^2}}}$$

where λ is the wavelength and α the convergent semi-angle of the incident electron beam, respectively, and ${\Delta}z$ in this study is approximately 76 nm. In the result, most of the rod-shaped arranged carbon support aggregates could be observed in an in-focus state without using the dynamic focus function. In general STEM image observation, a relatively large convergence semi-angle α is used to realize a small electron beam pot in order to improve spatial resolution, and the depth of focus is only a few nanometers in this condition (Eq. (2)). Because the information outrange of Δz degrades the transmission potential image of the whole specimen, the field of view for the thickness direction that can be observed practically is limited by Δz. In this study, a 7.5 mrad convergence semi-angle was selected to satisfy the spatial resolution, Δz (field of view for thickness direction), and the LAADF-STEM conditions described earlier.

It has also been reported that the dual-axis tomography method [32] is effective to reducing missing wedges. Although we attempted to observe specimens by this technique, we found that a specimen on a carbon microgrid support is not suitable for dual-axis tomography because the robust mesh skeleton prevents the penetration of electron beams under a high-tilt angle condition, and use of an elastic carbon film support TEM grid results in a non-linear increased background signal at a high tilt angle, which prevents accurate 3D reconstruction and segmentation. Therefore, we selected single-axis tomography with a high-tilt angle for this study, and performed the analysis using this technique.

In order to realize a minute 3D structure with nanometer resolution, fine image alignment was performed by the bead-tracking method [33] using a Pt catalyst particle (diameter: ∼3–5 nm). The final aligned image stacks had errors averaged over the image shift of under 0.8–2.0 pixels, i.e. <0.4 nm accuracy, which is about 10 times more accurate than when only the image correlation method is used. Tilt axis refinement was performed manually using several reconstructed HAADF orthogonal cross-section images. The ML-EM 3D reconstruction calculation method and its iteration number were decided based on an experimental consideration and past reports [34,35]. Among all the calculation methods available in software, such as weighted backprojection, simultaneous iterative reconstruction technique, and simultaneous algebraic reconstruction technique, this method was the least affected by the artifacts due to missing wedges in all the ADF image stacks.

### Segmentation scheme by *k*-means method

The image segmentation process is an important part of the tomography process. Essentially, the reconstruction image has grayscale brightness values originating from the projection ADF 2D images. In the 2D projection images, i.e. ADF 2D images, two brightness regions can be assumed, that is, the material and the vacuum area. However, in a 3D reconstructed image, artifact contrast exists in an intermediate brightness range between the two. The segmentation process in tomography is a process of extracting the material contrast. In general, the contrast of the material is extracted by setting a threshold value for brightness, but in many cases, manual fine-tuning by viewing the image is required because the same threshold value cannot be used every time due to the sample conditions, detector gain and other factors. This means that arbitrariness cannot be eliminated in this manual process. To realize a segmentation process without arbitrariness, we examined various automatic binarization methods such as the Otsu method [36], and found that it is necessary to perform segmentation of at least three values, namely, the vacuum state, the material and the artifact, indicating that a clustering method such as the *k*-means method is needed.

The *k*-means method is a type of unsupervised machine learning, and several studies have already applied this method to segmentation of various tomograms [37–39]. The *k*-means method that we used is a function in the Avizo software, but to solve the initial value problem of the *k*-means method, the initial clusters are arranged by random number generation in a reproducible manner [40] so that differences in the results due to initial-value dependence do not occur. The validity of this simple contrast enhancement process and *k*-means method-based segmentation process of the carbon and ionomer was verified quantitatively by the total volume amount ratio of the carbon support and ionomer, as shown in Table 2 (calculated I/C).

The spatial distribution validity of the ionomer in this method will be discussed in a subsequent publication that compares the electrochemical characterization related to the structural difference of the carbon support and the modification state of the ionomer.

### Thickness distribution of ionomer

**Fig. 5.**
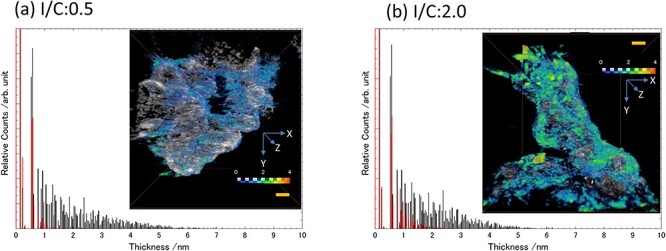
Calculated thickness of ionomer (left: I/C = 0.5, right: I/C = 2.0). The black and red plots represent the impregnated ionomer and the ionomer adhering to the outer side (unimpregnated ionomer), respectively. The inset images show the ionomer thickness distribution map. The gray contrast corresponds to the carbon support, and the scale bars correspond to 20 nm.

The thickness of the ionomer was calculated by the maximum diameter of a sphere inscribed inside the ionomer 3D structure. The detailed calculation method was described by Hilderbrand *et al.* [41]. In Fig. 5, the total ionomer and unimpregnated ionomer are indicated separately. In previous reports where the ionomer coating thickness was measured, the ionomer was identified by staining with a heavy element or other method, so the method proposed here corresponds to the total ionomer thickness value shown by the black line. The value of the red line corresponds to the commonly imagined ionomer coverage state, in which the ionomer covers the carbon support and the Pt particles exposed on the surface. The thickness map for I/C = 2.0 shows red regions with thicknesses >4 nm at some positions, indicating that the ionomer does not take the form of a uniform film, but rather displays flocculation that can include large spheres. However, the total thickness plot (black) shows no significant difference between I/C = 0.5 and 2.0 because it is normalized by the total pixel count of the ionomer. In contrast, the outside ionomer thickness of I/C = 2.0 shows an increment in the counts over the 1–2 nm region compared with I/C = 0.5. This result corresponds qualitatively to the decrease in the impregnation ratio in Table 2. That is, increasing I/C causes impregnation in the pores in the carbon support, but when the ionomer increases beyond the volume of pores that can be impregnated, the excess ionomer is considered to accumulate on the surface in a lump-like state, and in actual PEFC systems, this has been shown to reduce power generation performance by inhibiting oxygen diffusion [42,43].

Regarding the validity of the film thickness calculated using 3D data, the ionomer attached outside of the carbon support can be directly observed and measured by Cs-corrected MAADF-STEM images (Supplementary Fig. S2), to confirm the molecular chain of the ionomer without disturbance by the phase contrast, as in HRTEM images.

### Impregnation of ionomer into carbon support

**Fig. 6.**
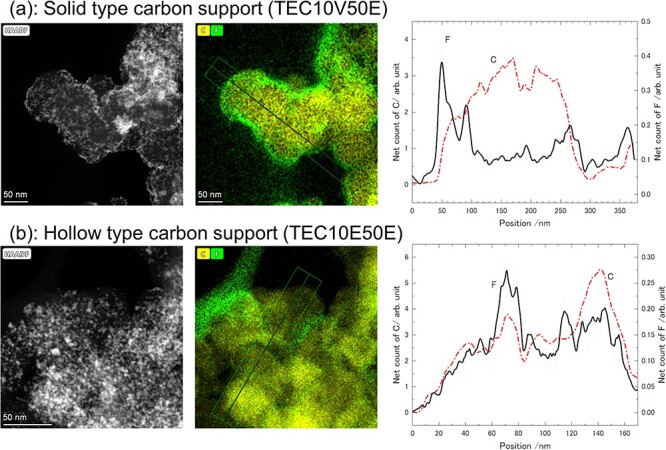
(Left) HAADF-STEM images, (center) EDX elemental mapping images, and (right) line profiles of net count values obtained from the position indicated by the arrows in the EDX elemental mapping images (line profiles were integrated by 51 pixel width). The upper and lower rows are the results for a solid-type carbon support (TEC10V50E) and a hollow-type carbon support (TEC10E50E), respectively.

Because the combined ADF-STEM tomography technique represents the spatial distributions of the components of the PEFC catalyst individually by different ADF information, the composition at the same coordinates can be expressed in 3D, as in EDX elemental mapping images. In this study, we demonstrated the impregnated state of the ionomer into a carbon support by 3D analysis. This impregnation state could also be verified by the EDX line analysis in Fig. 6. In particular, the ionomer does not impregnate into the solid-type carbon support, and the distribution of F (ionomer) covered the outer side of the C (carbon support). The line profile also shows the characteristic core-shell structure. In contrast, the distributions of C and F are overlapped on the hollow-type carbon support, which indicates impregnation. In the actual ionomer impregnation phenomenon, it is considered that the ionomer enters the pores or slit of the carbon support below the spatial resolution of EDX (<1 nm). The same is also true in tomography, and as a result, actual impregnation of the carbon support into the framework has not been confirmed, even by high-resolution STEM or other techniques (Supplementary Fig. S2).

### Degradation of ionomer due to electron beam irradiation

The PEFC catalyst also includes an ionomer as a key component for proton conductivity. This ionomer for PEFC application is one kind of perfluorosulfonic acid, such as Nafion. Like PTFE, the ionomer has (C–F)_2_ bonding as its polymer principal chain. As is well known, PTFE shows excellent chemical stability but poor resistance to radiation such as electron beam irradiation [44,45]. Therefore, the ionomer in the PEFC catalyst decomposes gradually as a result of observation and analysis in an electron microscope, and this eventually causes morphological change. This is a serious problem for tomography, which requires information from large numbers of frames taken from various directions at the same position.

This electron beam damage of the specimen is an unresolved problem of many years’ standing in electron microscopy observation of soft materials. Although the Cryo-TEM technique is one of the best solutions for suppressing electron beam damage in observation of soft materials, the cooling mechanism of the TEM specimen holder is complex and takes up considerable space around the specimen, thus restricting the tilting angles or shielding of the characteristic X‐ray from the specimen. For this reason, the Cryo-TEM technique is not suitable for tomography and/or EDX analysis.

**Fig. 7.**
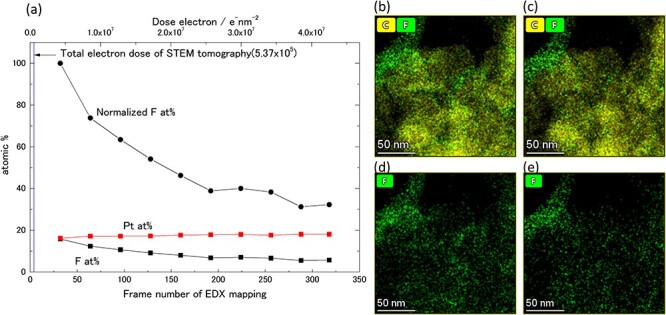
Change of EDX quantitative analysis data with series frame data acquisition. (a): Atomic fraction changes of F and Pt during EDX data acquisition. (b)–(e): extracted 32-frame integrated EDX elemental mapping images (net count) series EDX data. (b)–(d) and (c)–(e) were constructed using frames 1–32 (first 32 frames) and 286–318 (last 32 frames) of the integration data, respectively.

Therefore, in this study, we evaluated the degradation of the ionomer by electron beam irradiation at room temperature quantitatively by frame resolved EDX analysis. The irradiation beam conditions and details of the data acquisition setting are shown in the following Table 3, and results of the frame-resolved EDX analysis are shown in Fig. 7.

**Table 3.** Experimental conditions of data acquisition

	Unit	STEM tomography	EDX mapping
Pixel size	nm	0.19	0.37
Resolution	px	2048	512
Field of view	nm	381.3	188.4
Probe current	pA	3.75	145
Dwell time	μs	5	20
Frame time	s	20.97	5.24
Frame number		159	383
Electron dose per frame	e^−^/nm^2^	3.38E + 03	1.34E + 05
	e^−^/Å^2^	3.38E + 01	1.34E + 03
Total electron dose	e^−^/nm^2^	5.37E + 05	5.12E + 07
	e^−^/Å^2^	5.37E + 03	5.12E + 05

Figure 7a shows the decrease in F at% with respect to Pt at%, i.e. the amount of degradation of the ionomer response to electron irradiation. The X-ray counts of Pt per frame are considered to be constant regardless of the electron irradiation dose, and the normalized F at% from the Pt at% and F at% in each integrated frame was calculated. Figure 7b**–**e also show the EDX element mapping data constructed using the first 32 frames ((b) and (d)) and last 32 frames ((c) and (e)). These EDX element mapping data do not show any significant depletion of the ionomer due to electron irradiation, confirming that the ionomer spatial distribution itself, as shown in EDX elemental mapping data such as Fig. 2, shows correct results. However, the EDX quantification results show that the amount of ionomer in the last 32 frames is about half of that in the first 32 frames. Since the general EDX quantification calculation outputs the result of integrating all these frames, care must be taken in handing the values of the composition ratios.

On the other hand, the leftmost vertical line in Fig. 7a shows the total amount of electron irradiation of the developed combined ADF-STEM tomography method. As shown in Table 2, the total electron dose to acquire 159 series tilt STEM images was approximately only 4 frames of the EDX mapping analysis, indicating that the ionomer reduction was almost negligible and the decrease in the amount of ionomer (F) will be under the detection limit of EDX analysis. Although our result of the total electron dose amount is large compared to a past report [46] of cryo-electron tomography, no significant sample deformation was observed in the tomography or even the EDX mapping measurement results. While the reason for this is not clear, it is possible that once an electron beam is instantaneously irradiated at a location, there is an interval of several seconds to several tens of seconds before it is irradiated again, allowing the heat conditions caused by electron beam irradiation to moderate, and as a result, no significant deformation is observed.

## Conclusion

Nanoscaled direct observation of a PEFC catalyst without ionomer staining was achieved by the newly developed combined ADF-STEM tomography technique with quantitative 3D analysis. This method uses multiple ADF detectors with different electron beam collection angles to identify materials based on differences in signal intensity due to differences in the electron beam scattering capacity depending on the atomic number of the material. The tomography method is used to calculate a reconstructed arbitrary cross-sectional image, and the signals from each ADF detector are processed by arithmetical operations to construct images which emphasize compositional information, i.e. pure Z-contrast images without the effect of thickness. The *k*-means method was applied for segmentation from grayscale images, and quantitative analysis without arbitrariness was also demonstrated for items such as the I/C ratio and coverage thickness. The electron beam irradiation damage of the ionomer was evaluated by an EDX analysis, which showed that the dose amount of electrons through tomography acquisition was controlled to less than few frames of acquisition of EDX mapping, that is, degradation was suppressed to under the detection limit of EDX quantification. By optimizing the electron beam collection angle according to the element of interest, this technique can be applied not only to carbon and ionomers, as demonstrated in this study, but also to a variety of other materials with only slight differences in average atomic numbers, such as polymer alloys and composite materials.

## Supplementary Material

dfae002_Supp
