# Supplementary figures and images for: Three-dimensional nanostructure analysis of non-stained Nafion in fuel-cell electrode by combined ADF-STEM tomography
Source: Microscopy (Oxf). 2024 Jan 13;73(4):318–28. doi: 10.1093/jmicro/dfae002 (PMC11288185; doi:10.1093/jmicro/dfae002)

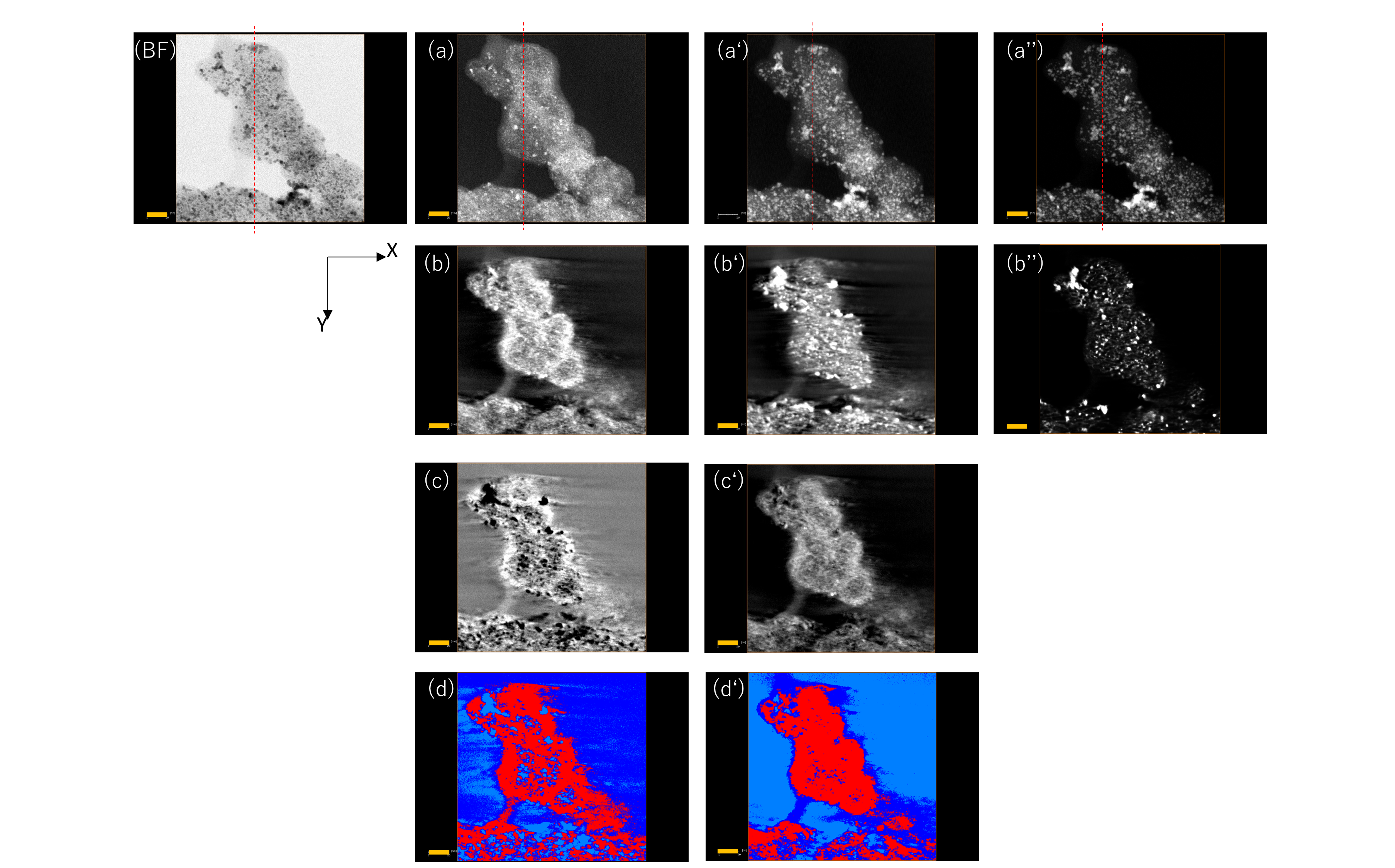

Supplement: dfae002_Supp [file dfae002_supp.zip › suppl_data/Fig-S1.tif]

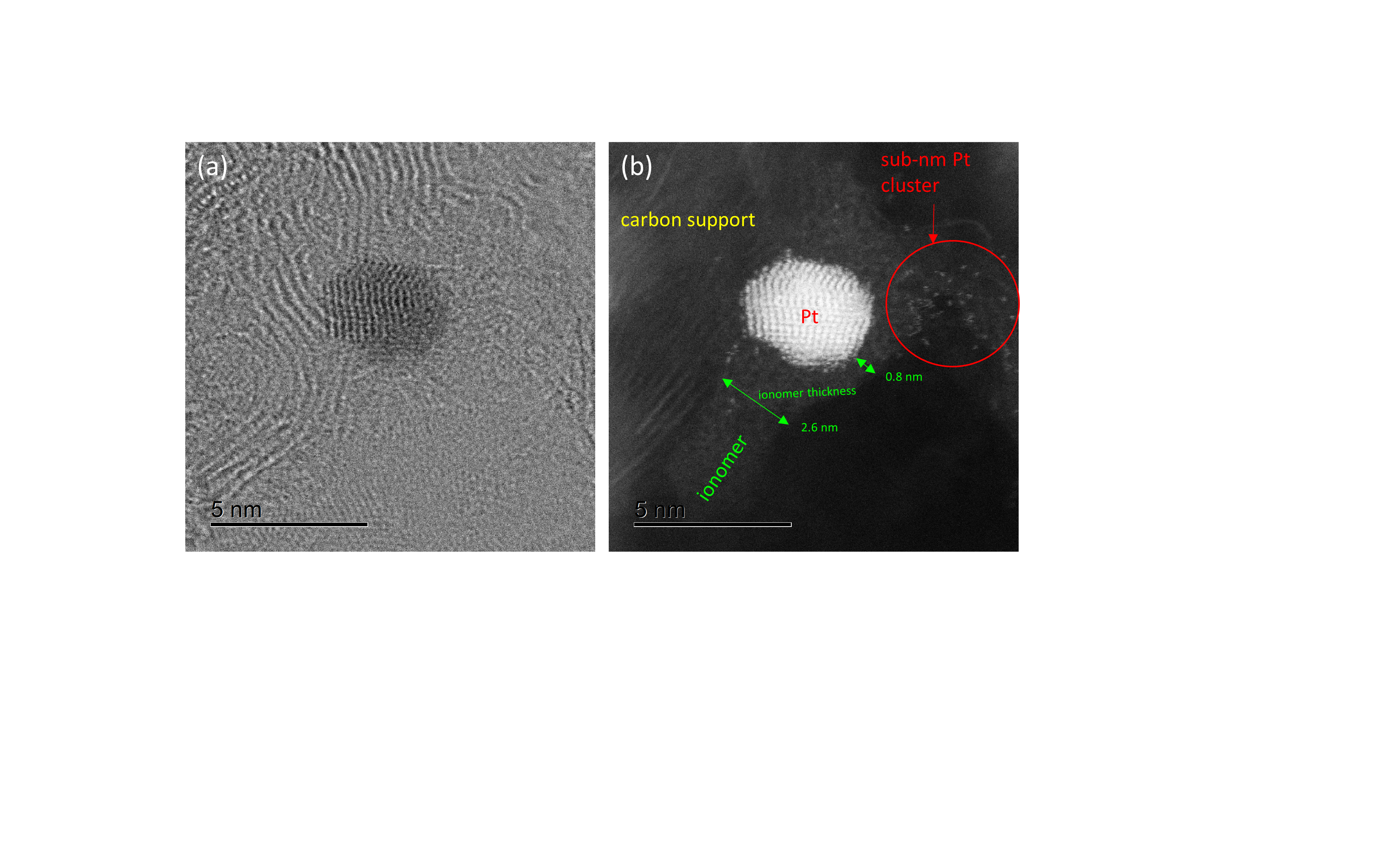

Supplement: dfae002_Supp [file dfae002_supp.zip › suppl_data/Fig-S2.tif]
